# *Campylobacter jejuni* acquire new host-derived CRISPR spacers when in association with bacteriophages harboring a CRISPR-like Cas4 protein

**DOI:** 10.3389/fmicb.2014.00744

**Published:** 2015-01-05

**Authors:** Steven P. T. Hooton, Ian F. Connerton

**Affiliations:** Division of Food Sciences, School of Biosciences, University of NottinghamLoughborough, UK

**Keywords:** *Campylobacter*, CRISPR, bacteriophage, Cas4, carrier state life cycle

## Abstract

*Campylobacter jejuni* is a worldwide cause of human diarrhoeal disease. Clustered Repetitively Interspaced Palindromic Repeats (CRISPRs) and associated proteins allow *Bacteria* and *Archaea* to evade bacteriophage and plasmid infection. Type II CRISPR systems are found in association with combinations of genes encoding the CRISPR-associated Cas1, Cas2, Cas4 or Csn2, and Cas9 proteins. *C. jejuni* possesses a minimal subtype II-C CRISPR system containing *cas1, cas2*, and *cas9* genes whilst *cas4* is notably absent. Cas4 proteins possess 5′-3′ exonuclease activity to create recombinogenic-ends for spacer acquisition. Here we report a conserved Cas4-like protein in *Campylobacter* bacteriophages that creates a novel split arrangement between the bacteriophage and host that represents a new twist in the bacteriophage/host co-evolutionary arms race. The continuous association of bacteriophage and host in the carrier state life cycle of *C. jejuni* provided an opportunity to study spacer acquisition in this species. Remarkably all the spacer sequences observed were of host origin. We hypothesize that *Campylobacter* bacteriophages can use Cas4-like protein to activate spacer acquisition to use host DNA as an effective decoy to bacteriophage DNA. Bacteria that acquire self-spacers and escape phage infection must overcome CRISPR-mediated autoimmunity either by loss of the interference functions leaving them susceptible to foreign DNA incursion or tolerate changes in gene regulation.

## Introduction

CRISPR systems constitute a rudimentary immune system for prokaryotes that enables them to survey, adapt and inactivate invading pathogens and parasites (Horvath and Barrangou, 2010). Three distinct CRISPR types are recognized (Types I–III), which comprise of various proteins and ribonucleoprotein complexes that concertedly mediate the capture of short DNA sequences (protospacers) from invasive genomes, such as those represented on bacteriophages (phages) and plasmids (Sorek et al., 2013; Spilman et al., 2013; Fonfara et al., 2014). Foreign DNA elements are incorporated into a CRISPR array as spacer DNA sequences flanked by conserved direct repeats. CRISPR-associated (*cas*) genes are frequently located within the immediate vicinity of an array, which encode proteins with endo/exonuclease activities required for protospacer acquisition. All CRISPR systems encode Cas1 DNA endonucleases and Cas2 RNA endonucleases that function to generate dsDNA protospacers of ~30–50 bp in size (Shah et al., 2013; Sorek et al., 2013). Upon integration into the array the spacers are expressed as small antisense CRISPR RNA molecules (crRNAs) that serve to guide proteins such as Cas3 (Type I CRISPR) and Cas9 (Type II CRISPR) to their intended targets during subsequent reinfection events (Marraffini, 2013). This process is termed CRISPR interference, the ultimate consequence of which is cleavage of the foreign DNA molecule thus protecting the bacterium/archaeon from the attempted infection event (Marraffini and Sontheimer, 2010).

Type II CRISPR systems are characterized by the presence of genes encoding various configurations of Cas1, Cas2, Cas4, and Cas9 proteins accompanied by a *trans*-activated CRISPR RNA (tracrRNA). Subtype II-A CRISPR systems found in *Streptococcus* spp. also contain the genes *csn2a*/*csn2b* (Fonfara et al., 2014). The minimal nature of Type II CRISPR systems requires that they use host RNase III during the biogenesis of crRNA molecules (Sampson and Weiss, 2013; Sorek et al., 2013). *Campylobacter jejuni* NCTC 11168 and *C. jejuni* PT14 are reported to contain subtype II-C CRISPR systems comprising of Cas1, Cas2, Cas9 proteins and a tracrRNA, whilst no homolog of Cas4 has been identified (Dugar et al., 2013; Brathwaite et al., 2013). Following Cas1/Cas2-mediated protospacer capture and subsequent crRNA biogenesis, a dsRNA molecule comprised of crRNA and tracrRNA (complementary base-paired at the 3′-end of the antisense crRNA) serves to target Cas9 endonuclease to the invading DNA element. Whilst not present in every characterized Type II CRISPR system, it has recently been shown that Cas4 proteins of subtype II-B systems possess 5′-3′ exonuclease activity and are involved in the adaptation process of CRISPR immunity (Zhang et al., 2012; Lemak et al., 2013). The generation of ssDNA 3′-ends by Cas4 produces potentially recombinogenic 3′-overhangs that allow strand invasion-mediated incorporation of the captured protospacer into the CRISPR array. Cas4 proteins have a number of signature motifs that are observed to be structurally similar to the AddB exonuclease component of the AddAB helicase/exonuclease complex found in *Bacillus subtilis* (Saikrishnan et al., 2012). The crystal structure of Cas4 SsO0001 from *Sulfolobus solfataricus* has recently been reported to be a decameric toroidal structure formed by the interactions of five Cas4 dimers (Lemak et al., 2013). Each Cas4 protein contains a 4Fe-4S binding cluster and a bound Mn^2+^ molecule is predicted to occupy the active site of each monomer. Cas4 proteins contain four absolutely conserved cysteine residues that form the 4Fe-4S cluster (Zhang et al., 2012; Lemak et al., 2013) with conserved domains and motifs present in a wide range of RecB exonucleases to which they are related (Sisáková et al., 2008).

Due to the intricate associations that have guided the evolutionary pathways of bacteria and their phages, it is of little surprise that phages have evolved mechanisms to evade targeting by host CRISPR systems. The complexity of these systems range from single nucleotide polymorphisms in protospacer-adjacent motif (PAM) sequences of phage 2972 that circumvent CRISPR-mediated immunity by *Streptococcus thermophilus* DGCC7710 (Sun et al., 2013), to the specific prophage-encoded anti-CRISPR proteins found in Mu-like phages that infect *Pseudomonas aeruginosa* PA14 (Bondy-Denomy et al., 2013). In a role reversal *Vibrio cholerae*-specific *Myoviridae* phages contain a complete Type I-F CRISPR system that overcomes the anti-phage activity of a host encoded pathogenicity island (Seed et al., 2013). Here we report a unique phage-encoded Cas4-like protein in *Campylobacter* phages. Bioinformatic analysis suggests that the protein is structurally similar to Cas4 (SsO0001) of *S. solfataricus* and contains four conserved cysteines associated with 4Fe-4S cluster formation, and conserved RecB exonuclease motifs. Interestingly, protein homologs are found distributed throughout all Class II (Cp220likevirus) and Class III (Cp8unalikevirus) *Campylobacter* phages that compose the *Eucampyvirinae* (Javed et al., 2014). The Type II-C CRISPR systems of *C. jejuni* notably lack Cas4 proteins. The absence of *cas4* may hinder spacer acquisition including bacteriophage defense but the recoupling of the *cas4* component results in the incorporation of self-derived *Campylobacter* DNA with the potential to modify host gene expression and evolution within the species as a response to these changes.

## Materials and methods

### Bacteria and bacteriophages

*C. jejuni* PT14 was routinely grown on horse blood agar at 42°C under microaerobic conditions (85% N_2_, 5% O_2_ and 10% H_2_) for 18 h as previously described (GenBank accession CP003871; Brathwaite et al., 2013). Class III bacteriophages CP8 and CP30A were isolated and propagated as described previously (respective GenBank accessions KF148616 and JX569801; Loc Carrillo et al., 2005). Carrier state *Campylobacter* cultures PT14CP8CS and PT14CP30ACS were formed and propagated as described previously (Siringan et al., 2014).

### DNA sequencing

Genomic DNAs were prepared from *C. jejuni* PT14 and carrier state cultures propagated on blood agar plates using GenElute™ (Sigma-Aldrich, Dorset, UK), from which 5 to 7 million 100 bp reads were generated using MiSeq technology operating in paired-end mode (Illumina, San Diego, California, USA) and imported into CLC Genomics Workbench 6.0 for analysis.

### Bioinformatics

Database searches were conducted using the BLAST suite of programs at NCBI (Camacho et al., 2009). Protein sequence alignments and phylogenetic trees were calculated using ClustalW2 (McWilliam et al., 2013). Protein structure models were created and superimposed using the Phyre2 server (Kelley and Sternberg, 2009).

### Transcriptome analysis

Total RNAs were extracted from three independent early-log phase cultures of *C. jejuni* PT14 growing in Mueller-Hinton broth (Oxoid, Basingstoke, UK), using TRIzol® Max™ with Max™ Bacterial Enhancement Reagent (Invitrogen, Paisley, UK) and depleted of rRNA using Ribo-Zero™ rRNA removal kit for Gram negative bacteria (Epicentre Biotechnologies, Madison, Wisconsin, USA). Aliquots were incubated with Terminator™ 5′-monophosphate-dependent exonuclease (TEX) (Epicentre Biotechnologies) to remove processed RNAs. cDNA libraries were prepared with TruSeq™ RNA (Illumina) and adapter indexed and run on Illumina MiSeq operating in single-read mode. Raw sequence reads were imported into CLC Genomics Workbench 6.0 for analysis. Differential expression was determined using the RPKM expression values in conjunction with Baggerly's test statistic (Baggerly et al., 2003) using a False Discovery Rate (FDR) correction (Benjamini and Hochberg, 1995).

## Results

### Campylobacter bacteriophages harbor CRISPR Cas4-like proteins

**Figure 1:**
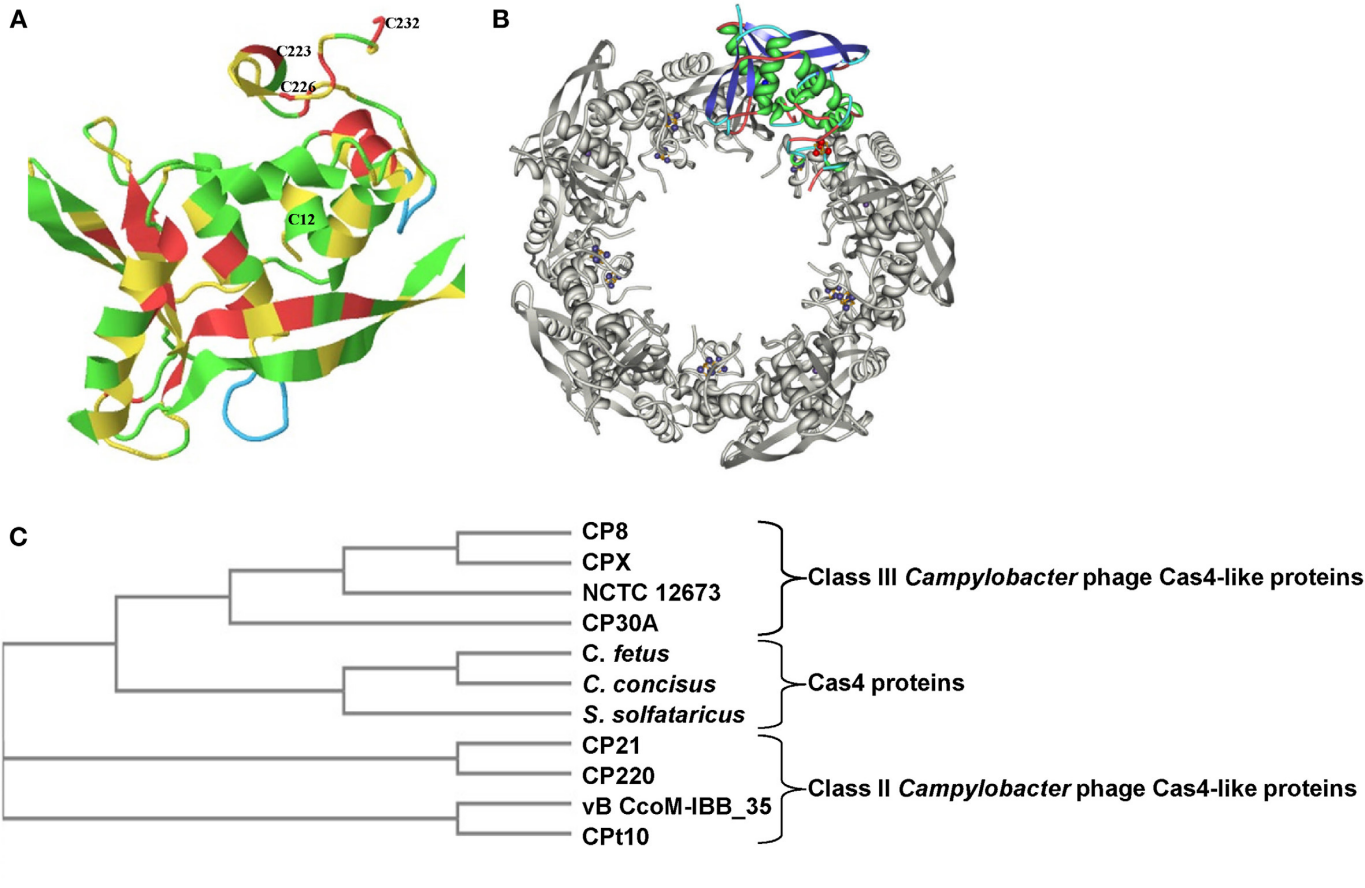
**Structural conservation of the C-terminal 4Fe-4S cluster-forming cysteines of Cas4-like proteins from *Campylobacter* phages. (A)** The predicted structure of the bacteriophage CP8 Cas4-like protein monomer from Phyre2 indicating the amino acid positions of the four conserved cysteine residues that form a 4Fe-4S cluster; **(B)** Arrangement of the monomer structures within the decameric toroidal Cas4 protein (SsO0001) of *S. solfataricus* showing positions of the 4Fe-4S clusters (13); **(C)** Phylogenetic analysis using a neighboring-joining tree without distance correction (ClustalW2) revealing lated family of proteins where Class III phage Cas4-like proteins cluster with *C. fetus, C. concisus* and SsO0001.

Bioinformatic analysis of the genome of *Campylobacter* phage CP8 revealed the presence of a hitherto unannotated gene of 732 bp that initiates using a GUG codon and encodes a putative 243 amino acid Cas4-like protein (predicted molecular weight 28.8 kDa.). The gene is highly conserved amongst other Class III *Campylobacter* phages (CPX, CP30A, NCTC 12673, and CP81) although BLASTN reveals no other significant similarities outside of the phage class. Translation reveals diverged protein homologs exist in Class II *Campylobacter* phages CP220, CP21, and Cpt10 as well as many bacterial proteins. The domain architecture of CP8 Cas4-like protein is strikingly similar to other proteins recognized as belonging to the Cas4 family with a number of signature motifs distributed throughout the protein. Bacteriophage CP8 Cas4-like protein can be structurally aligned with Cas4 protein (SsO0001) of *Sulfolobus solfataricus* (99.75% confidence). Figure 1A shows the absolute conservation of cysteine residues C12, C223, C226, and C232 that correspond with a highly ordered 4Fe-4S iron binding domain. A RecB EXXXXXL motif I (ECFRQCKL) is located at residues 11–18 of the CP8 Cas4-like protein, the first cysteine in this motif corresponding with the N-terminal C12 residue of the 4Fe-4S cluster. A PD(D/E)XK (PDDDK) nuclease domain is observed to span residues 111–121, a QXXXY motif (QLALY) is found at residues 156–160, whilst the three C-terminal conserved cysteines are located from residues 223–232. *In silico* mutational analysis of the conserved cysteines indicates the importance of these amino acids with regards to the function of the 4Fe-4S cluster and the integrity of the quaternary decameric structure (Figure 1B). In Class II and Class III *Campylobacter* phages, the four cysteine residues forming the Fe-S cluster are highly conserved despite significant divergence in total amino acid composition. ClustalW2 alignments using Class II and Class III *Campylobacter* phage Cas4-like amino acid sequences show that the EXXXXXXL motif, PD(D/E)XK nuclease domain, QXXXY motif, and the four cysteine residues are all conserved in these proteins. A phylogenetic tree demonstrating the relationship between the *Campylobacter* phage Cas4-like proteins and Cas4 proteins of *S. solfataricus, C. concisus* UNSW2 and *C. fetus* 03-427T is presented in Figure 1C. Class III *Campylobacter* phages cluster as a sub-branch of the Cas4 proteins, whilst Class II *Campylobacter* phages form a distinct branch.

### The CRISPR array of *C. jejuni* PT14

The genome sequence of *C. jejuni* PT14 contains a minimal type II-C CRISPR array organized in four 36 bp direct repeats flanking three 30 bp spacer elements. Beginning 144 bp upstream of the terminal direct repeat are the genes encoding the CRISPR-associated proteins Cas2 (A911_07325), Cas1 (A911_07330), and Cas9 (A911_07335). BLASTN analysis of the 36 bp direct repeats shows that they are highly conserved and present in a large number of *C. jejuni* CRISPR systems. The spacers within the *C. jejuni* PT14 CRISPR array show no obvious matches to foreign DNA, and instead harbor nucleotide sequences that can be mapped to the PT14 chromosome. Table 1 summarizes the sequence matches observed for spacers 1 and 2, whereas only short regions of homology (<12 nucleotides) can be identified between spacer 3 and the PT14 chromosome. Spacers 1 and 3 are also represented in the CRISPR arrays of *C. jejuni* strains R122, R35, and FO14.

**Table 1:** **Native and acquired spacer DNAs of the CRISPR array of *C. jejuni* PT14**.

**Spacer sequence^a^**	**Frequency^b^**	**PAM^c^**	**Target strand^d^**	**Match**	**Gene product**	**Locus tag**
**NATIVE *C. JEJUNI* PT14 SPACERS 1-3**						
ATAATTTCTAA**TTTCATTTAT**A**ACCTTTCA**	1	AAG	+	(18/30)PT14	Peptidoglycan-associated lipoprotein Omp18	A911_00540
TAGTAGCT**AAGAATAAAATAAGAA**ACACTG	1	TTC	+	(16/30)PT14	Apolipoprotein N acyltransferase	A911_05300
TAGTAGCTAAGAAT**AAAATAAGAAACACT**G		CCA	+	(15/30)PT14	*IleS*—isoleucyl tRNA synthetase	A911_05135
**GTTGGAATGCTT**AAGCAGGGGTGGAGTGAA	1	CAT	−	(12/30)PT14	Di/tripeptide transporter	A911_03195
**RE-ACQUIRED SPACER 3 (CS30)**						
GTTGGAATGCTTAAGCAGGGGTGGAGTGAA	6.1 × 10^−2^					
ATTGGAATGCTTAAGCAGGGGTGGAGTGAA	1.8 × 10^−3^					
GATGGAATGCTTAAGCAGGGGTGGAGTGAA	9.1 × 10^−4^					
GGTGGAATGCTTAAGCAGGGGTGGAGTGAA	9.1 × 10^−4^					
GTTAGAATGCTTAAGCAGGGGTGGAGTGAA	9.1 × 10^−3^					
G**TTTGAATGCTTAAG**CAGGGGTGGAGTGAA	9.1 × 10^−^4	TTT	−	(14/30)PT14	Hypothetical protein	A911_02745
GTTGGGATGCTTAAGCAGGGGTGGAGTGAA	9.1 × 10^−4^					
GTTGGAGTGCTTAAGCAGGGGTGGAGTGAA	9.1 × 10^−4^					
G**TTGGAAAGCTTAA**GCAGGGGTGGAGTGAA	9.1 × 10^−4^	TTT	−	(13/30)PT14	ATP-binding subunit ClpA protease	A911_05365
**GTTGGAACGCTTA**AGCAGGGGTGGAGTGAA	1.8 × 10^−3^	TTT	−	(13/30)PT14	Intergenic	A911_04960
GTTGGAATACTTAAGCAGGGGTGGAGTGAA	9.1 × 10^−4^					
GTTGGAATTCTTAAGCAGGGGTGGAGTGAA	9.1 × 10^−4^					
GTTGGAATGCTCAAGCAGGGGTGGAGTGAA	1.8 × 10^−3^					
GTTG**GAATGCTTG**AGCAGGGGTGGAGTGAA	9.1 × 10^−4^	AAC	−	(13/30)PT14	Citrate synthase	A911_08105
GTTGGAATGCTTA**G**GCAGGGGTGGAGTGAA	9.1 × 10^−4^					
GTTGGAATGCTTAAACAGGGGTGGAGTGAA	9.1 × 10^−4^					
GTTGGAATGCTTAAGTAGGGGTGGAGTGAA	1.8 × 10^−3^					
GTTGGAATGCTTAAGCGGGGGTGGAGTGAA	1.8 × 10^−3^					
GTTGGAATGCTTAAGCAAGGGTGGAGTGAA	3.2 × 10^−3^					
GTTGGAATGCTTAAGCAGAGGTGGAGTGAA	2.7 × 10^−3^					
GTTGGAATGCTTAAGCAGTGGTGGAGTGAA	1.8 × 10^−3^					
GTTGGAATGCTTAAGCAGGAGTGGAGTGAA	9.1 × 10^−4^					
GTTGGAATGCTTAAGCAGGGGAGGAGTGAA	9.1 × 10^−4^					
GTTGGAATGCTTAAGCAGGGGCGGAGTGAA	9.1 × 10^−4^					
GTTGGAATGCTTAAGCAGGGGTGGGGTGAA	9.1 × 10^−4^					
GTTGGAATGCTTAAGCAGGGGTGGACTGAA	9.1 × 10^−4^					
GTTGGAATGCTTAAGCAGGGGTGGATTGAA	9.1 × 10^−4^					
GTTGGAATGCTTAAGCAGGGGTGGAGCGAA	1.8 × 10^−3^					
GTTGGAATGCTTAAGCAGGGGTGGAGGGAA	9.1 × 10^−4^					
GTTGGAATGCTTAAGCAGGGGTGGAGTAAA	9.1 × 10^−4^					
GTTGGAATGCTTAAGCAGGGGTGGAGTGAG	9.1 × 10^−4^					
**CS8 ACQUIRED NAIVE SPACERS**						
TAAAAATTTAAGCCCGCAAAGTCAAATTTC	5.5 × 10^−4^	ATC	−	(30/30)PT14	Hemin binding protein	A911_07785
TGCTTAAATCCCCAAGTTTTTCTAAAAATT	5.5 × 10^−4^	GAG	+	(30/30)PT14	Conserved membrane protein	A911_02540
TTATCTCCTTCTCCATCTCCATTATTATAA	5.5 × 10^−4^	TGC	−	(30/30)PT14	Hemolysin activation/secretion protein	A911_04705
TAAAATCTTTAAAATATTCTAAATTTTTTT	1.1 × 10^−3^	CAC	+	(30/30)PT14	PseE motility associated protein	A911_06495
TCAAATACTTTTATGCTTTATGATACATTT	1.1 × 10^−3^	CAA	−	(30/30)PT14	Capsular polysaccharide biosynthesis	A911_06909
CGCTAAGTTTTACAACTACTCAATTTTTAG	5.5 × 10^−4^	CCG	−	(29/30)PT14	L-Lactate permease	A911_00360
TAAGATCTTCCAAGCTATGGCTTGAAATTT	5.5 × 10^−4^	TAC	−	(29/30)PT14	tRNA mo(5)U34 methyltransferase	A911_04710
TT**GTAGCGTCTAAAATTTCATTTTTTTCAA**	5.5 × 10^−4^	TTA	+	(28/30)PT14	Isocitrate dehydrogenase	A911_02585
**CS30 ACQUIRED NAIVE SPACERS**						
TAGAGCTTGTTTATAACGGGATAGTTTATT	9.1 × 10^−4^	TCT	+	(30/30)PT14	ADP-heptose-LPS heptosyltransferase II	A911_05560
CAATAATCAATCTCAACTCCACTCCTATCA	4.5 × 10^−4^	TCC	+	(30/30)PT14	CRISPR-associated protein Cas8c/Csd1	A911_05640
CTTTGCTTTTTGGATAATCAGAGAGGAAGA	4.5 × 10^−4^	CAA	−	(30/30)PT14	argF ornithine carbamoyltransferase	A911_04795
TGGCTTCATATTTGATATAAGTACCACGAT	4.5 × 10^−4^	ATG	+	(30/30)PT14	Putative tungsten ABC-transport system	A911_07415
GGCATTATTGAGCTGGTGTTTGCTCTTTTG	9.1 × 10^−4^	CAG	+	(30/30)PT14	Conserved hypothetical protein	A911_00810
GCTGGTGTTTGCTCTTTTGTTTTTTTGTGA	4.5 × 10^−4^	TCA	+	(30/30)PT14	Conserved hypothetical protein	A911_00810
ATAGAGCTTGTTTATAACGGGATAGTTTTT	4.5 × 10^−4^	CTT	+	(29/30)PT14	ADP-heptose-LPS heptosyltransferase II	A911_05560
CTGATACTCAACTATTTTAAAGGAATTCCA	4.5 × 10^−4^	CAT	+	(29/30)PT14	Translation elongation factor P	A911_02685
TGGCTCTAAAACTCCGCTCATATAAACCAA	4.5 × 10^−4^	CTT	+	(29/30)PT14	Anaerobic C4-dicarboxylate transporter	A911_00415
**TCTATATCTTTTTTAAAATTTTT**A**AT**CT**A**A	9.1 × 10^−4^	AAT	+	(26/30)PT14	ADP-heptose-LPS heptosyltransferase I	A911_05490
“		ATC	+	(21/30)PT14	Molybdate ABC-transporter	A911_01445
“		CTT	+	(21/30)PT14	Spermidine/putrescine ABC-transporter	A911_03575
“		GAA	+	(21/30)PT14	Glycosyltransferase	A911_05505
TTT**TA**C**CGTCTAAAATTTCATTTTTTTCAA**	4.5 × 10^−4^	TTA	+	(26/30)PT14	Isocitrate dehydrogenase	A911_02585
**AAGGGGTTGGCTTTTTGGATTCT**TTTT**TAA**	4.5 × 10^−4^	TAA	+	(26/30)PT14	nusG Transcription antiterminator	A911_02310
**TTTTTTCTTTATTTTAAA**AT**TT**TT**A**A**T**CTA	4.5 × 10^−4^	TTA		(22/30)PT14	Intergenic	A911_03110

a *Partial spacer sequence matches to the PT14 chromosome are indicated in bold. Nucleotide substitutions in the acquired spacer 3 sequences are underlined*.

b *The frequency with respect to invariant spacer 1 sequence reads mapping to the CRISP array*.

c *PAM is the observed proto-spacer adjacent tri-nucleotide sequence*.

d *Target strand indicates whether the spacer binds the coding (+) or non-coding (−) DNA strands of PT14*.

**Figure 2:**
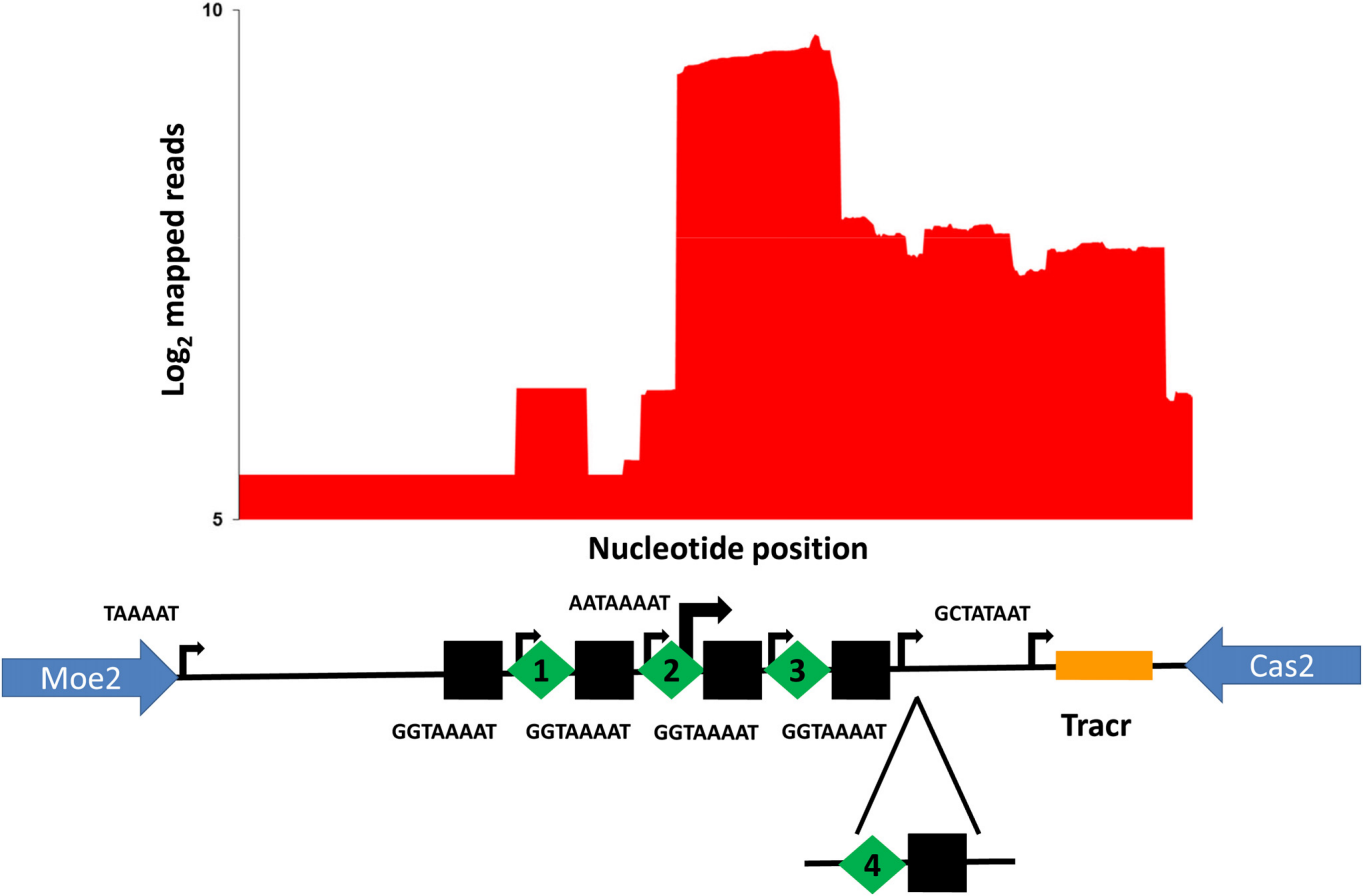
**Organization and transcription of the CRISPR-array of *C. jejuni* PT14**. The CRISPR array is located between *moe*2 (A911_07320) and *cas*2 (A911_07325). The plot shows log_2_ cDNA read counts from libraries constructed from TEX treated *C. jejuni* PT14 RNA mapped to the CRISPR array. The relative positions of direct repeats are indicated as black squares, the spacers as green diamonds (spacers 1–3) and the tracrRNA in gold. Transcription start sites are marked by black arrows where the greater transcription arising from spacer 2 indicated by the width of the arrow. The −10 nucleotide motifs with respect to the transcription start sites are indicated adjacent to the arrows for the spacer 2 and the tracrRNA, and those common to the direct repeats below the black squares. The position of spacer acquisition in the carrier state cultures is indicated below (spacer 4).

The organization of the *C. jejuni* PT14 CRISPR array is similar to other *C. jejuni* in that the CRISPR array is located following the reading frame of *moaA* (A911_07320) with a leader sequence that contains a putative −10 nucleotide transcriptional start site (5′-TAAAAT-3′) before the start of pre-crRNA synthesis located 116 nucleotides upstream of the initial direct repeat of the CRISPR array. As reported previously each direct repeat in the CRISPR array contains an independent promoter such that a single RNase III/tracrRNA dependent processing event is required to create the mature crRNA (Dugar et al., 2013). The direct repeats contain the motif 5′-GGTAAAAT-3′ that resembles an extended −10 nucleotide transcriptional start site signal that results in a transcriptional start site 4 nucleotides into the following spacer (Figure 2). However, RNAseq data generated from rRNA depleted RNA indicates transcription within the *C. jejuni* PT14 CRISPR array also arises within the second spacer using a similar -10 nucleotide motif of 5′-TAAAAT-3′. Treatment with 5′-monophosphate-dependent exonuclease (TEX) further revealed that this transcription start site dominates transcription within the CRISPR region, some 15-fold greater than the transcription start sites distal to the direct repeats (Figure 2). The dominant start site is located 7 nucleotides before direct repeat 3, which would favor the expression of the 3′-end of spacer 2 and spacer 3.

### The acquisition of spacers in carrier state cultures

The carrier state of *C. jejuni* is characterized by the association of host bacteria with bacteriophages in an equilibrium state such that the viable count to the phage titer ratio are maintained upon serial propagation (Siringan et al., 2014). Carrier state cultures PT14CP8CS and PT14CP30ACS were passaged five times using mass inoculums on blood agar plates to represent at least 100 generations of *Campylobacter* in association with the corresponding Class III bacteriophages (CP8 or CP30A). Genomic DNAs were prepared from these cultures with similarly treated wild type *C. jejuni* PT14, from which 5 to 7 million sequence reads were collected for each sample using the MiSeq platform. DNA sequence reads that contained flanking direct repeats were selected and examined for nucleotide changes in the intervening spacer sequences or for the acquisition of new spacer sequences within the *C. jejuni* PT14 CRISPR array. New spacer sequences were identified from the carrier state cultures but not PT14. The carrier state populations of PT14CP8CS and PT14CP30ACS contained bacteria that had extended the CRISPR array by either acquisition of an additional copy of spacer 3 or naive spacer sequences (indicated as spacer 4 in Figure 2). In all cases the spacer acquisitions were positioned adjacent to spacer 3 toward the distal end of the array, and were of a uniform length of 30 nucleotides as are the native spacers. Nucleotide changes were centered on the additional spacer 3 DNA sequence and flanking direct repeats whilst spacer sequences 1 and 2 remained fixed. Table 1 documents the nucleotide sequence changes observed in the additional copy of spacer 3 and the naive sequences acquired by the carrier state cultures. Notably all the naive spacer DNA sequences could be mapped to the host genome sequence and were not represented in either of the co-propagating bacteriophage genomes.

## Discussion

The presence of a Cas4-like protein in *Campylobacter* bacteriophages represents another unexpected twist in the co-evolutionary pathway with their hosts. The maintenance of the protein throughout Class II and Class III *Campylobacter* phages suggests that the activity confers a selective advantage. Whilst significant divergence in overall amino acid sequence identity is observed between the two phage classes, the conserved cysteine residues and signature motifs of Cas4 proteins are all present. This poses the question as to how the Cas4-like proteins in *Campylobacter* bacteriophages operate, whether these proteins have a defined role in phage infection and/or enable the phages to exert control over their host bacteria. Type II CRISPR-Cas systems show flexibility in their arrangement with Cas9 being divorced from Cas2 in 31 of 103 reported examples (Makarova et al., 2011). Further evidence for the modular assembly of Type II CRISPR-Cas systems has been proposed for Csn2 that has an analogous role to Cas4 in the integration spacer sequences in subtype II-A systems. Phylogenetic analysis of Cas1 and Cas9 proteins reveals the subtype II-A branch to be embedded within subtype II-C protein sequences, which implies that the subtype II-A system is a derivative of the subtype II-C, and that Csn2 has been acquired later (Chylinski et al., 2014). Subtype II-B systems contain Cas4 orthologs, however the addition of phage encoded Cas4 to the subtype II-C system does not phylogenetically resemble a subtype II-B system. Subtype II-B systems are reported to originate from type I CRISPR-Cas systems with respect to their Cas1, Cas2, and Cas4 protein sequences and CRISPR repeats (Chylinski et al., 2014). The configuration of a subtype II-C with the phage encoded copy of Cas4 represents a new combination. The ability of phage encoded Cas4 to modify spacer element acquisition in a host subtype II-C system could potentially allow the phage influence the content of the CRISPR array in an attempt to evade or subvert interference.

The carrier state of *C. jejuni* provided an opportunity to examine the operation of the CRISPR-Cas system in the presence of Class III bacteriophage carrying Cas4. Carrier state cultures derived from *C. jejuni* PT14 that propagate either bacteriophage CP8 or CP30 were selected on the basis of their ability to maintain near constant viable count to bacteriophage titer ratios, and on the observed genomic stability of the bacterium and bacteriophage (Siringan et al., 2014). As a pre-requisite we examined transcription within the CRISPR array of *C. jejuni* PT14 using RNASeq data. The CRISPR array of *C. jejuni* PT14 contains 4 direct repeats interspersed with 3 spacer sequences of 30 nucleotides, where each spacer has the potential for independent expression based on promoter sites located within the preceding direct repeat. This arrangement is consistent with the organization and transcription of other CRISPR-containing *C. jejuni* strains (Brathwaite et al., 2013). However, spacer 2 of *C. jejuni* PT14 contains an additional strong promoter that results in strong expression of the 3′-end of spacer 2 and spacer 3. Pairing of tracrRNA with the preceding direct repeat would leave an 8 nucleotide overhang from the spacer 2/direct repeat boundary of 5′-AACACUGG-3′. Whether 8 nucleotides is a sufficient guide to locate the Cas9-containing complex is not clear but the sequence has the potential to pair with 10 sites within the PT14 genome, 1 within CP8 (encoding the DNA end protector) and 2 with CP30 (encoding the DNA end protector and peptidase). Maturation of the major transcript originating within spacer 2 by RNase III cleavage produces abundant spacer 3 crRNA.

Type II CRISPR-Cas systems are generally reported to bind and cleave DNA through the action of Cas9 (Jinek et al., 2012), and it is clear that targeting of chromosomal sequences will be detrimental leading to either cell death or genome rearrangement (Vercoe et al., 2013). However, examples exist whereby partial base-pairings to crRNAs do not result in DNA cleavage (Jinek et al., 2012) but rather have the potential to alter gene expression. For example, partial pairing of crRNA to Mu-like prophage gene 42 of *Pseudomonas aeruginosa* does not result in cell death or confer phage resistance but results in Cas-interference dependent modulation of swarming behavior and biofilm formation that is likely mediated by crRNA binding mRNA to cause antisense inhibition (Zegans et al., 2009; Cady et al., 2012). Two of the native spacer sequences of *C. jejuni* PT14 have partial sequence matches that could influence the expression of cell surface components and therefore bacteriophage recognition and entry. The nucleotide sequences of spacer 1 and the 5′-end of spacer 2 have the potential to pair respectively with mRNA encoding outer membrane lipoprotein Omp18 (A911_00540) and apolipoprotein *N*-acyltransferase (A911_05300). Transcription of the gene encoding apolipoprotein *N*-acyltransferase is barely detectable in *C. jejuni* PT14, which would influence the presentation of acylated lipoproteins and enable the strain to evade recognition by Toll-like receptors (TLR) 1/2 and any resulting pro-inflammatory response. TLR 1/2 signaling is a significant component of the pro-inflammatory response to *C. jejuni* by human cell lines (Al-Sayeqh et al., 2010). However, the antisense crRNAs to Omp18 and apolipoprotein *N*-acyltransferase are weakly expressed in comparison with spacer 3 that has 15-fold greater expression but limited self-recognition and almost no sequence similarity to Class III bacteriophages CP8 and CP30.

We initiated this study to examine the influence of bacteriophage encoded Cas4 on spacer acquisition. The presence of bacteriophages in the carrier state indeed resulted in spacer acquisition but all the spacers were of host origin. Bacteriophage protospacers were not targeted despite continued replication of the bacteriophage. Whether bacteriophage protospacers are preferentially not recognized or do not become fixed in the CRISPR array is unclear but the properties of bacteriophage DNA by which it could evade protospacer selection include base modification or the presence of protective protein-DNA interactions. The function of Cas4 in spacer acquisition is the generation of ssDNA 3′-ends that enable strand invasion-mediated incorporation into the CRISPR array. The most abundant acquired spacer represents the reintegration of an additional copy of spacer 3. The presence of flanking sequences in the paired sequence reads enabled the new spacer 4 to be located at the distal end of the array in what appears to be a relatively frequent process (1 in 16 relative to the read count recorded for spacer 1). This is in contrast to *in vivo* studies with *Streptococcus thermophiles*, which indicate that spacers are added at the leader end of the CRISPR array and lost from the distal end such that new spacers are added at the expense of old ones in response to new challenges (Deveau et al., 2008). Similarly it is reported that spacer acquisition occurs at the leader end of *Escherichia coli* BL21 upon over-expression Cas1 and Cas2, and where the disruption of spacer transcription does not to prevent acquisition (Yosef et al., 2012). Using this system mutations introduced into the first repeat were observed to be propagated during spacer acquisition indicating that the proximal repeat acts as the template for subsequent generations of the repeat sequence (Yosef et al., 2012). However, the first direct repeat of *C. jejuni* CRISPR-arrays contains a 5′-terminal nucleotide substitution compared to all the subsequent repeats that was not propagated during the acquisition process. Inspection of the acquired spacer 4 sequences and flanking direct repeats reveals sequence reads containing single nucleotide substitutions that extend proximal to the major transcription start site located seven nucleotides within spacer 2, and distal to the final direct repeat. Before and after this location the sequence reads are conserved. Degeneration of the direct repeat is a feature of subtype II-B CRISPR systems that also use Cas4 in their integration mechanism. It is tempting to suggest that the resumption of sequence conservation marks the boundaries on the resolution of the recombination complex responsible for introducing the new spacer and that the single nucleotide substitutions mark repair sites following Cas4-mediated strand invasion.

The deep sequencing strategy employed in this study allows even rare molecular events to be recorded but provides no evidence as to the fitness of the resulting bacteria or whether they even survive. By definition the CRISPR array is a target for crRNA but since wild type *C. jejuni* array sequences are maintained, these must be either exempt or actively surveyed and repaired to insure against CRISPR-directed DNA cleavage. Expansion of the CRISPR array with an existing spacer may benefit from pre-existing protective mechanisms; however the acquisition of ectopic chromosomal sequences is likely to increase the risk of CRISPR-mediated autoimmunity (Stern et al., 2010). Self-derived spacer-acquisition has been reported to occur repeatedly, and in the majority of cases lead to cell death (Yosef et al., 2012). We have observed the acquisition of naive self-spacer sequences in the CRISPR arrays of carrier state *C. jejuni*, and unlike the native spacer DNA sequences many of these show near or complete sequence identity with host chromosomal genes. The high degree of sequence conservation may reflect the recent acquisition of these sequences, and as stated above we cannot determine how long the bacteria carrying these spacers survive. It is possible that CRISPR-mediated autoimmunity is maintained in this context as an altruistic response to persistent bacteriophage infection but if this were the case it does not explain why the bacteriophage should maintain a functional component of the system. It is possible that the bacteriophage mobilize host-spacer acquisition as a decoy to prevent phage DNA acquisition, and therefore the expression of Cas4 is an anti-CRISPR measure. Escape of CRISPR-mediated autoimmunity can occur through mutation of components of the host CRISPR-Cas system including the *cas* genes, spacers, repeats or protospacer targets if not essential (Vercoe et al., 2013). Loss of a functional CRISPR-Cas would leave the bacteria open to horizontal gene transfer since natural transformation is common in the genus, with the long term consequence of genome homogenization and erosion of genome features that would formerly differentiate the bacteria. It is reported that *C. coli* are subject to introgression from *C. jejuni* as both inhabit the intestinal tract of commercial poultry (Sheppard et al., 2008). CRISPR-mediated autoimmunity could therefore be profoundly directing the shape of evolution in these species.

The fate of the infected host during the lytic life cycle of the bacteriophage would have no impact on the reproductive success of the bacteriophage. However, under the specialized circumstances that give rise to the carrier state life cycle, then the acquisition of host spacers could be detrimental to the continued survival of the host and the bacteriophage. In these experiments we cannot deduce if self-spacer acquisition can provoke mutation but we note the multiple occurrence of the same spacer sequence and independent targeting of proto-spacers within the same gene or genes of related function amongst the naive spacer sequences acquired. These observations may be due to bias in the adaptation mechanism or due to bacterial replication and selection post-spacer acquisition. It is also possible the expression of the crRNAs themselves are altering bacterial gene expression, which would provide a reason for the multiple acquisition of spacers targeting proto-spacers in the same genes or those of related function and another reason for the bacteriophage to maintain Cas4 in order to subvert and/or reprogram the host bacteria to pave the way to the carrier state under conditions which otherwise would lead to low replicative success of the bacteriophage. Antisense transcription is not abnormal in *C. jejuni* with a recent high resolution survey reporting that 45% of the transcriptional start sites direct antisense transcription in multiple strains (Dugar et al., 2013). *C. jejuni* can certainly tolerate antisense expression but may also use the process as a form of gene regulation since campylobacters are generally noted for their minimal quota of regulatory genes. In this context we note the acquisition of spacers that produce crRNAs that could act as antisense RNA inhibitors/modulators, for example the CRISPR-associated protein Cas8c/Csd1 and three independent spacers targeting ADP-heptose-lipo-oligosaccharide heptosyltransferase systems. Heptosyl residues form a link that enables decoration of lipo-oligosacharides in *C. jejuni*, the absence of which would prevent further carbohydrate addition that includes Gal-GalNac-NeuAc moieties that are also associated with human ganglioside types involved in the GM1 or GD1 molecular mimicry associated with Guillian-Barre syndrome and severe gastro-enteritis (Godschalk et al., 2007; Mortensen et al., 2009). It has been suggested that lipo-oligosacharide sialylation represents a defense mechanism that protects *C. jejuni* from bacteriophage infection (Louwen et al., 2013), which would be abrogated by preventing the expression of ADP-heptose-lipo-oligosaccharide heptosyltransferase.

## Author contributions

Steven P. T. Hooton and Ian F. Connerton performed the experimental and computational analyses, and wrote the manuscript.

### Conflict of interest statement

The authors declare that the research was conducted in the absence of any commercial or financial relationships that could be construed as a potential conflict of interest.
